# Association between dietary soy prevention of fetal alcohol spectrum disorder and normalization of placental insulin and insulin-like growth factor signaling networks and downstream effector molecule expression

**DOI:** 10.36922/gpd.3113

**Published:** 2024-06-13

**Authors:** Fusun Gundogan, Ming Tong, Suzanne M. de la Monte

**Affiliations:** 1 Department of Pathology and Laboratory Medicine, The Warren Alpert Medical School of Brown University, Providence, Rhode Island, United States of America; 2 Department of Pathology and Laboratory Medicine, Women and Infants Hospital of Rhode Island, Providence, Rhode Island, United States of America; 3 Liver Research Center, Department of Medicine, Rhode Island Hospital, Providence, Rhode Island, United States of America; 4 Departments of Pathology and Laboratory Medicine, Neurology, and Neurosurgery, Rhode Island Hospital, Providence, Rhode Island, United States of America

**Keywords:** Aspartyl-asparaginyl-β-hydroxylase, Notch, mRNA, Polymerase chain reaction

## Abstract

Chronic prenatal alcohol exposure causes fetal alcohol spectrum disorder (FASD), often associated with impaired placentation and intrauterine growth restriction. Ethanol’s inhibition of insulin and insulin-like growth factor Type 1 (IGF-1) signaling compromises trophoblastic cell motility and maternal vascular transformation at the implantation site. Previous studies have demonstrated that dietary soy effectively normalizes placentation and fetal growth in an experimental model of FASD. The studies were extended to better understand the mechanisms underlying soy’s beneficial effects. Pregnant Long Evans rats were pair-fed with isocaloric liquid diets containing either 0% or 36% caloric ethanol from gestation day (GD) 6. The protein source in the diets consisted of either casein (standard and control) or soy isolate. On GD19, placentas were harvested to measure mRNA levels corresponding to major components of the insulin/IGF-1 pathway, as well as aspartyl-asparaginyl-β-hydroxylase (ASPH), Notch, and HES, which play critical roles in placentation. Chronic gestational ethanol exposure in rats fed diets containing casein significantly reduced the expression of insulin, insulin receptor, *Igf1*, IGF-1 receptor (*Igf1r*), insulin receptor substrate Type 1 (*Irs1*), *Irs2*, *Asph*, and *Hes1*. In addition, ethanol significantly decreased ASPH protein expression. Dietary soy mitigated most of these effects and further enhanced signaling by upregulating *Igf2*, *Igf2r*, *Irs1*, *Irs2*, *Irs4*, *Notch*, and *Hes1* in rats chronically exposed to ethanol relative to corresponding control samples. The protective effects of dietary soy in FASD act at the mRNA level and positively impact pathways imperative for normal placentation and fetal development. Gestational dietary soy may provide an effective means of preventing FASD in vulnerable populations.

## 1. Introduction

Fetal alcohol spectrum disorder (FASD) is a significant public health concern, driven by socioeconomic, mental health, and cultural disparities that contribute to heavy alcohol consumption during pregnancy. In addition to characteristic craniofacial abnormalities, FASD features birth and neurodevelopmental defects, intrauterine growth restrictions (IUGR), postnatal growth limitations, and increased fetal mortality rates. Placentation, encompassing placental maturation, growth, and implantation, is integral for optimizing the fetal environment, ensuring ample nutrient delivery and waste product removal. The occurrence and severity of FASD are partially rooted in placental dysfunction, mediated by impairments in placentation. Previous studies have established a link between FASD-mediated impairments in placentation and reduced signaling through insulin and insulin-like growth factor (IGF) pathways.^1^ These pathways regulate the expression and function of aspartyl-asparaginyl-β-hydroxylase (ASPH), which plays critical roles in regulating cell motility and invasion necessary for effective placentation.^2–5^ Heavy alcohol consumption during pregnancy compromises ASPH’s functions, leading to disruptions in critical cellular processes.^6^ Mechanistically, ASPH catalytically hydroxylates and activates Notch,^7,8^ which, in turn, upregulates transcription of hairy and enhancer of split-1 (HES1).^7,9^ Notch is pivotal in regulating a broad array of cellular functions during development, including growth, motility, and maturation.^10^ The inhibitory effects of ethanol have been well-documented.^9^

Potential preventive and treatment measures for FASD should take into consideration the intricate interactions among maternal factors, placental reactions, and the diverse fetal responses.^11^ Addressing problems arising from insulin/IGF resistance due to impaired signal transduction presents a promising avenue. Earlier research has demonstrated the efficacy of peroxisome-proliferator-activated receptor (PPAR) agonists in preventing or reducing the disease-related effects of dysregulated insulin/IGF signaling in experimental models of chronic alcohol exposure, obesity, or nitrosamine administration.^12–17^ PPAR agonists act by bypassing poorly responsive cell surface receptors and stimulating gene expression at the level of transcription within the nucleus.^18,19^ However, practical and health-related uncertainties regarding the administration of PPAR agonist medications to pregnant women warrant exploration into alternative approaches. Investigating whether the insulin-sensitizing effects of natural products, such as soy or bioactive isoflavones (daidzein and genistein),^20^ could counteract the adverse effects of developmental alcohol exposures is of particular interest. Recent experiments along these lines have yielded promising results, suggesting the potential of soy-based interventions to mitigate neurodevelopmental deficits associated with FASD.^21–23^ In a comprehensive model of FASD, dietary soy was found to effectively mitigate alcohol-mediated impairments in placentation and neurodevelopmental features.^1^ However, further mechanistic research is warranted to elucidate the underlying mechanisms and inform future clinical and population-based applications. The present study builds on our earlier efforts by examining the effects of alcohol and dietary soy on the mRNA expression of upstream insulin/IGF signaling pathway molecules, including *Asph*, *Notch1*, and *Hes1*. This investigation aims to assess the degree to which dietary soy mediates its protective effects on placentation at the mRNA level by modulating gene expression.

## 2. Materials and methods

### 2.1. Materials

Qiazol reagent, EZ1 RNA universal tissue kit, QuantiTect SYBR Green polymerase chain reaction (PCR) master mix, and the BIO Robot Z1 were procured from Qiagen Sciences Inc (USA). The AMV first strand cDNA synthesis kit was obtained from Roche Diagnostics Corporation (USA). Enzyme-linked immunosorbent assay (ELISA) plates and the ELISA plate washer were sourced from Thermo Scientific Nunc (USA). Horseradish peroxidase (HRP)-conjugated secondary antibodies and the soluble fluorophore (Amplex Red) were supplied by Invitrogen (USA).

### 2.2. *In utero* ethanol exposure model

The Lifespan Institutional Animal Care and Use Committee (IACUC) of Rhode Island Hospital approved the experimental use and treatment of Long Evans rats for this research. On gestation day (GD) 6, following timed mating as previously described,^1^ we initiated feeding with isocaloric liquid diets (BioServ, USA). Control diets contained 0% ethanol. The experimental diets comprised 36% caloric ethanol or 8.2% vol/vol. The protein sources consisted of either 100% casein, which was considered a control due to its standard inclusion in rodent diets, or 100% organic soy protein isolate, which was considered an experimental. Consequently, four study groups were designated as follows: (i) CC for control-casein, wherein the rats were fed ethanol-free diets with casein as the protein source; (ii) CS, corresponding to ethanol-free diets with soy as the protein source; (iii) EC, in which the rats were fed ethanol-containing diets with casein as the protein source; and (iv) ES, corresponding to ethanol-containing diets with soy as the sole protein source. Eight placentas were randomly selected from each group for analysis. GD6 was selected as the start time for ethanol feeding because earlier exposures cause excessive fetal loss and impair placentation.^24,25^ The dams’ food intake, behavior, and body weights were monitored daily, as previously reported.^1^

### 2.3. Quantitative reverse transcriptase-PCR analysis

Quantitative reverse transcriptase-PCR (qRT-PCR) analysis was employed to measure insulin (*Ins*), *Igf1*, *Igf2*, insulin receptor (*Insr*), *Igf1r*, *Igf2r*, insulin receptor substrate type 1 (*Irs1*), *Irs2*, *Irs4*, *Asph*, *Notch1*, and *Hes1* mRNA transcripts using gene-specific primer pairs through the methodology previously described.^7^ The PCR primers were designed using MacVector 10 software (MacVector, Inc., USA), and their target specificities were confirmed through the NCBI-BLAST (National Center for Biotechnology Information-Basic Local Alignment Search Tool). Results were analyzed using the Mastercycler ep realplex instrument and software (Eppendorf AG, Germany). The relative abundance of each mRNA transcript was expressed as the calculated ratio of specific mRNA to 18S rRNA. All assays were performed in triplicate.

### 2.4. ELISAs

Placental tissue homogenates, prepared in radioimmunoprecipitation assay (RIPA) buffer containing protease and phosphatase inhibitors,^26,27^ were used to measure immunoreactivity to ASPH utilizing rabbit polyclonal antibodies. Protein concentrations were determined using the BCA assay. Direct binding ELISAs were conducted with 50 ng protein per sample.^9,21,28^ Immunoreactivity was detected using HRP-conjugated secondary antibody and Amplex Red soluble fluorophore.^26,28^ Fluorescence intensity was measured (Ex 530/Em 590) in a SpectraMax M5 microplate reader (Molecular Dynamics, USA). Negative control reactions were performed with primary, secondary, or both antibodies omitted. Between steps; the wells were rinsed three times with Tris-buffered saline (TBS) containing 0.05% Tween 20 (TBST) using a Nunc ELISA plate washer.^28^

### 2.5. Statistical analysis

Data corresponding to levels of gene expression or immunoreactivity are illustrated using boxplots and whiskers to depict the data distributions along with the mean (horizontal bar), 95% confidence interval limits (upper and lower limits of the boxplots), and range (upper and lower stems). Inter-group comparisons were conducted through one-way analysis of variance (ANOVA) followed by *post hoc* Tukey tests of significance. Statistical analysis was performed using the GraphPad Prism 10.2 software (USA). The threshold for statistical significance was set at *P* ≤ 0.05. For calculated *P*-values falling between 0.05 and 0.10, the statistical outcome was interpreted as indicative of a trend.

## 3. Results

While it is established that soy offers antioxidant and insulin-sensitizing benefits, the mechanisms implicated in normalizing placentation and fetal development vis-à-vis continued chronic high-level ethanol exposures have not been thoroughly evaluated. Herein, we investigated the potential effects of ethanol and dietary soy on insulin/IGF pathway gene expression, as previous studies indicated that chronic ethanol exposures can significantly modulate the expression of mRNA transcripts encoding proteins critical to signal transduction.^7^ Moreover, if the previously reported experimental responses to ethanol and dietary soy in placentas and fetuses were mediated by chronic alterations in gene expression, it would be necessary to ascertain measures that could ensure future therapeutic interventions favorably impact long-term pathophysiological processes responsible for sustained impairments in intracellular signaling. To achieve this, we measured mRNA levels corresponding to the insulin and IGF polypeptides and receptors, including *Ins*, *Igf1*, *Igf2*, *Insr*, *Igf1r*, *Igf2r*, *Irs1*, *Irs2*, *Irs4*, *Asph*, *Notch1*, and *Hes1*.

### 3.1. Insulin and insulin growth factor mRNA expression

**Figure 1.**
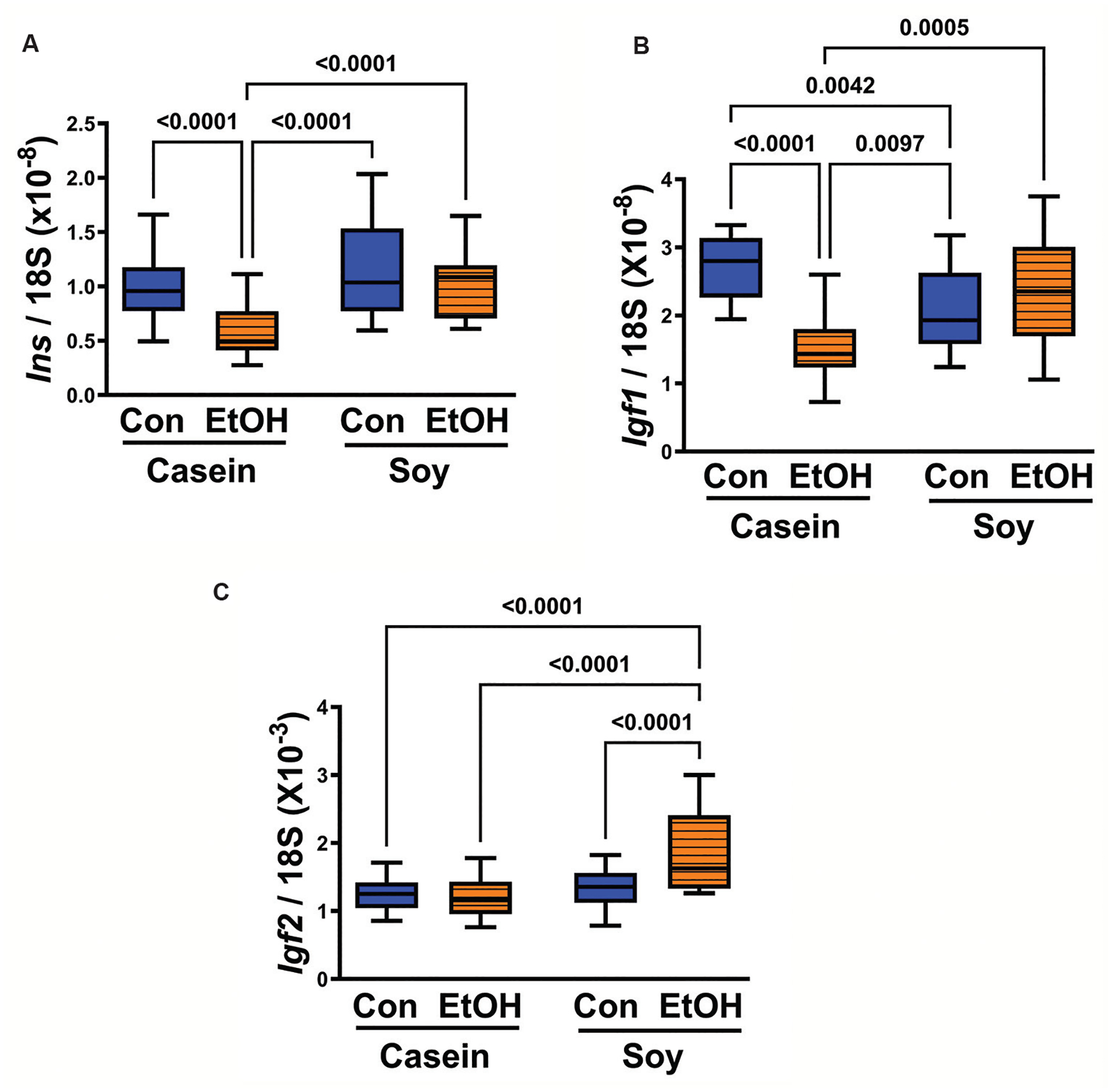
Effects of chronic gestational exposure to ethanol and dietary soy isolate on (A) *Ins*, (B) *Igf1*, and (C) *Igf2* mRNA expression in placental tissue. Reverse-transcribed RNA was polymerase chain reaction amplified using gene-specific primer pairs. Transcript abundance was calculated relative to 18S rRNA. Inter-group statistical comparisons were conducted using one-way analysis of variance (see Table 1) with *post hoc* Tukey tests. The *P* ≤ 0.05 are considered statistically significant. Notes: Con: Control; EtOH: Ethanol.

One-way ANOVA revealed significant inter-group differences in the levels of insulin (*P* < 0.0001), *Igf1* (*P* < 0.0001), and *Igf2* (*P* < 0.0001) expression (Table 1). Notably, *Igf2* exhibited markedly higher expression levels compared to *Igf1*, with insulin ranking second across all groups (Figure 1). Ethanol exposure induced a significant reduction in insulin (*P* < 0.0001) (Figure 1A) and *Igf1* (*P* < 0.0001) (Figure 1B) mRNA expression when standard casein served as the dietary protein source. However, there was no significant impact observed on *Igf2* expression (Figure 1C). Substituting dietary soy for casein effectively mitigated ethanol’s inhibitory effects on insulin (Figure 1A) and *Igf1* (Figure 1B) mRNA levels, normalizing them to comparable levels with the CC and CS groups while significantly surpassing expression in the ES group. Furthermore, dietary soy intake significantly elevated *Igf2* expression in the ES group compared to all other groups (*P* < 0.0001) (Figure 1C).

### 3.2. Insulin and insulin growth factor receptor expression

**Figure 2.**
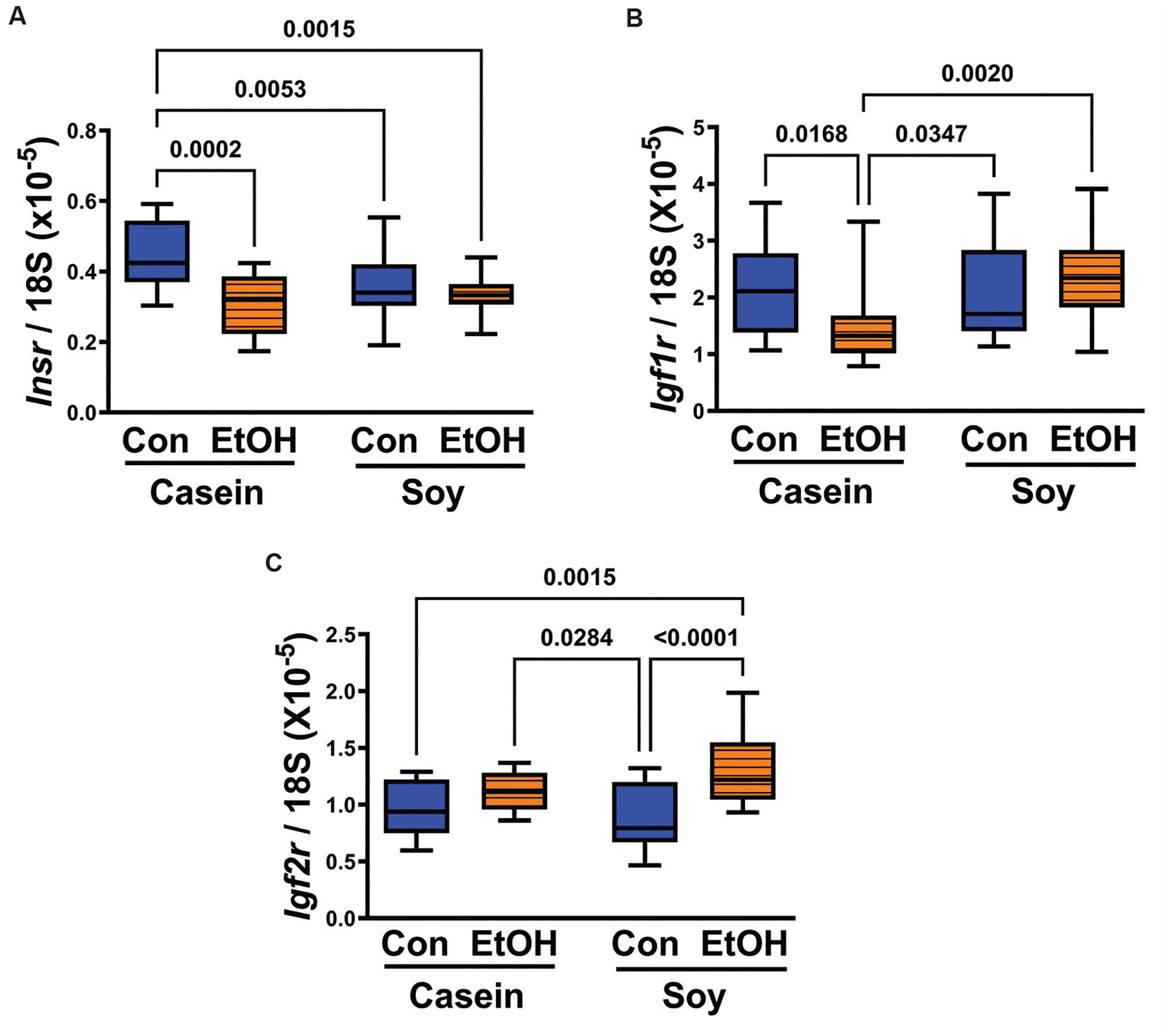
Comparisons of chronic gestational exposure to ethanol and dietary soy versus casein effects on (A) *Insr*, (B) *Igf1r*, and (C) *Igf2r* mRNA levels in placental tissue. Boxplots depict relative mRNA transcript abundance calculated from the ratio of specific polymerase chain reaction-amplified products (mRNAs) to 18S rRNA. Inter-group statistical comparisons were made by one-way analysis of variance (see Table 1) with *post hoc* Tukey tests. The *P* ≤ 0.05 are considered statistically significant. Notes: Con: Control; EtOH: Ethanol.

*Insr* (*P* = 0.0015), *Igf1r* (*P* = 0.014), and *Igf2r* (*P* = 0.0006) expression exhibited significant variations among the experimental groups, as revealed by one-way ANOVA (Table 1). Among these receptors, *Igf1r* demonstrated the highest abundance, followed by *Igf2r*, with *Insr* ranking third in expression levels (Figure 2). *Post-hoc* Tukey tests further elucidated significantly reduced *Insr* (*P* = 0.0002) (Figure 2A) and *Igf1r* (*P* = 0.0168) (Figure 2B) mRNA levels in the EC group compared to the CC group. Conversely, the expression of *Igf2r* mRNA remained consistent between EC and CC groups (Figure 2C). Replacing casein with dietary soy resulted in similar mean mRNA levels of *Insr* and *Igf1r* in both CS and ES groups. However, there was a notable elevation in *Igf2r* expression in the ES group relative to the other three groups (Figure 2C), mirroring, in part, the response observed in IGF-2 hormone responses (Figure 1C). Dietary soy correlated with lower levels of *Insr* but comparable levels of *Igf1r* in the CS and ES groups compared to the CC group. In contrast, *Igf1r* expression levels were similar across CS, ES, and CC groups, all of which exhibited higher expression levels compared to the EC group (Figure 2B).

### 3.3. Insulin receptor substrate expression

**Figure 3.**
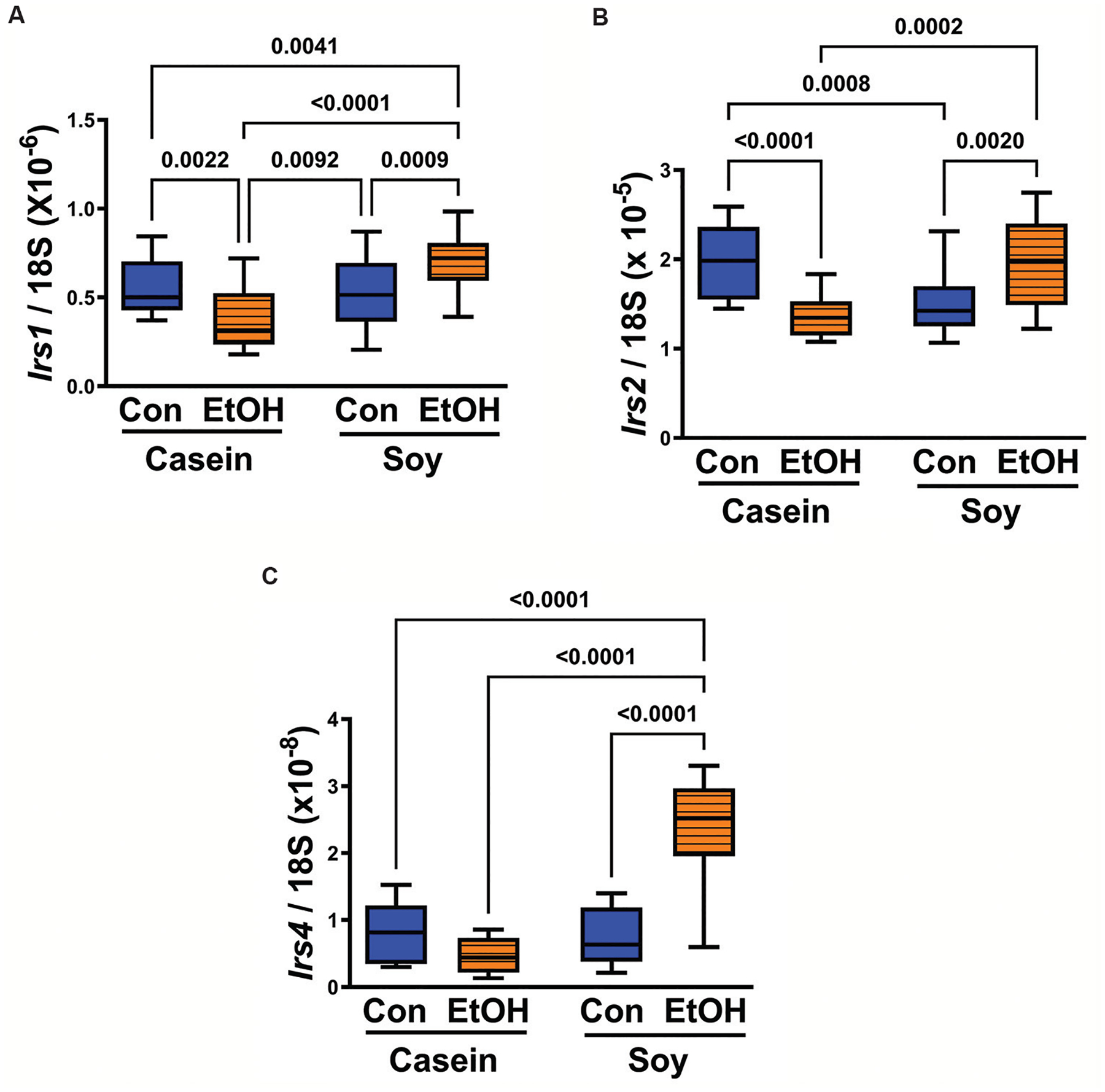
Effects of chronic gestational ethanol exposure in combination with dietary soy versus casein on the relative abundance of (A) *Irs1*, (B) *Irs2*, and (C) *Irs4* mRNA levels in placental tissue. Boxplots depict the calculated ratios of specific polymerase chain reaction-amplified products (mRNAs) to 18S rRNA. Inter-group statistical comparisons were conducted using one-way analysis of variance (see Table 1) followed by *post hoc* Tukey tests. The *P*-values ≤0.05 are considered statistically significant. Notes: Con: Control; EtOH: Ethanol.

One-way ANOVA analysis revealed significant inter-group differences in *Irs1*, *Irs2*, and *Irs4* expression (Table 1). Among these insulin receptor substrate proteins, *Irs2* emerged as the most abundant, followed by *Irs1*, with *Irs4* exhibiting the lowest expression levels (Figure 3). In rats subjected to chronic gestational exposure to ethanol while maintained on liquid diets containing casein as the primary protein source, a significant reduction in expression was observed for the two predominant insulin receptor substrate forms, *Irs1* (Figure 3A) and *Irs2* (Figure 3B). Conversely, control placentas from dams fed with soy isolate (CS) instead of casein exhibited comparable levels of *Irs1* (Figure 3A) and *Irs4* (Figure 3C), but demonstrated reduced expression of *Irs2* (*P* = 0.0008) (Figure 3B) relative to the CC group. The observed reduction in *Irs4* was modest and did not attain statistical significance (Figure 3C). Dietary soy intake did not exert significant effects on mRNA levels of *Irs1* or *Irs4* when comparing CS to CC groups. However, it significantly reduced the mean expression level of *Irs2* (*P* = 0.0008). In contrast, soy consumption prevented ethanol-induced reductions in insulin receptor substrate expression measured in the EC group, elevating *Irs1*, *Irs2*, and *Irs4* above levels measured in the CS and EC groups. In addition, *Irs1* and *Irs4* expression were significantly elevated in the ES group relative to the other three groups, while *Irs2* levels were normalized compared to the CC group.

### 3.4. Chronic gestational ethanol and dietary soy effects on placental *Asph*, *Notch1*, and *Hes1* expression

**Figure 4.**
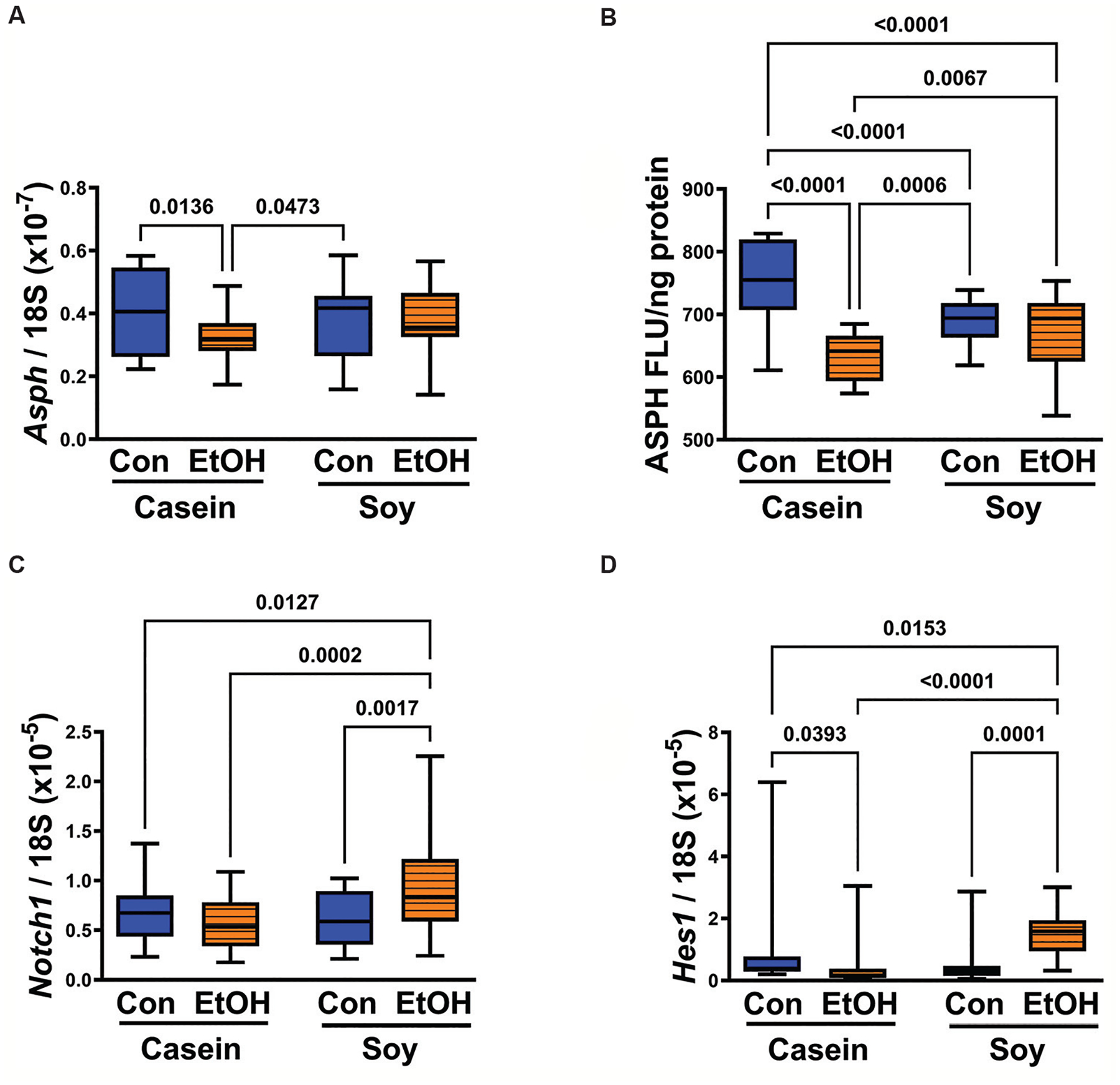
The inhibitory effects of ethanol on (A) *Asph* mRNA expression level and (B) ASPH immunoreactivity, as well as on (D) *HES1* mRNA expression level, and the stimulatory effects of dietary soy on (A) *Asph* mRNA expression level and (B) ASPH immunoreactivity, as well as on (C) *Notch1* and (D) *Hes1* mRNA expression levels. *Asph, Notch1*, and *Hes1* mRNA expression levels were measured using qRT-PCR analysis, with results normalized to 18S rRNA. ASPH immunoreactivity was measured using duplex ELISA, with immunoreactivity normalized to protein content. Inter-group statistical comparisons were conducted using one-way analysis of variance (Table 2) followed by *post hoc* Tukey tests. The *P* ≤ 0.05 are considered statistically significant. Notes: Con: Control; EtOH: Ethanol. Abbreviations: ASPH: Aspartyl-asparaginyl-β-hydroxylase; ELISA: Enzyme-linked immunosorbent assay; qRT-PCR: Quantitative reverse transcriptase polymerase chain reaction.

One-way ANOVA analysis indicated a statistical trend in *Asph* mRNA expression and a statistically significant difference in ASPH immunoreactivity (Table 2). *Post hoc* Tukey tests revealed that in the casein groups, ethanol significantly reduced *Asph* mRNA levels (*P* = 0.0136) (Figure 4A) and immunoreactivity (*P* < 0.0001) (Figure 4B) compared to corresponding controls. In contrast, in the soy groups, both control and ethanol-exposed groups exhibited similar levels of *Asph* mRNA (Figure 4A) and ASPH immunoreactivity (Figure 4B). Furthermore, *Asph* mRNA levels in the CS and ES groups were comparable to those in the CC group, whereas ASPH immunoreactivity levels, though elevated relative to the EC group, were reduced relative to the CC group (Figure 4B).

*Notch1* mRNA expression varied significantly among the groups, as indicated by one-way ANOVA analysis (*P* = 0.0013) (Table 2). Ethanol did not significantly inhibit *Notch1* expression. Instead, the main response was a significant elevation of *Notch1* expression in the ES group compared to the other three groups (Figure 4C). *Hes1* mRNA levels also varied significantly among the groups (*P* < 0.0001) (Table 2). *Post hoc* Tukey tests revealed significantly lower levels of *Hes1* in the EC group compared to the CC group, and higher levels in the CS group compared to the other three groups (Figure 4D).

## 4. Discussion

In an earlier companion study, we demonstrated that experimental chronic gestational exposure to ethanol led to increased fetal loss, IUGR, placentation abnormalities, and fetal morphogenic attributes reminiscent of FASD.^1^ These ethanol-induced effects were associated with altered expression of insulin, IGF, and downstream signaling proteins and phospho-proteins.^1^ The shifts in phospho-protein levels correspond with the net dynamic results of kinase and phosphatase activities, along with the expression levels of corresponding signaling molecules. However, we observed that replacing casein with soy in the diets diminished or eliminated many of the alcohol-associated impairments in insulin/IGF signaling in the placenta, reduced the occurrences of fetal loss, and prevented characteristic FASD-related morphometric developmental abnormalities.^1^ Importantly, the normalization of placentation and fetal growth was linked to the insulin-sensitizing and antioxidant effects of soy, which enhanced signaling through the insulin and IGF-1 receptors and downstream through Akt pathways in placental trophoblast.^1^

The overall goal of the present study was to investigate the mechanisms by which prenatal alcohol exposure impairs insulin and IGF signaling, and how soy mitigates these effects by characterizing the relevant effects on gene (mRNA) expression. Conceptually, ethanol could adversely impact intracellular signaling through insulin/IGF pathways by altering protein stability, turnover, and phosphorylation state through the regulation of kinase or phosphatase activities. Although we used the same previously described model, this study focused on insulin/IGF signaling through IRS pathways, primarily by measuring mRNA expression. In addition, since ASPH is a downstream target of insulin/IGF, plays a critical role in trophoblast motility and invasion required for implantation, and has been shown to be inhibited by ethanol in other FASD studies,^6^ we examined ASPH’s mRNA and protein expression to corroborate the ethanol-induced alterations in insulin/IGF signaling and further evaluate the insulin-sensitizing effects of dietary soy in our model.

Previous studies of rat placental tissue demonstrated the expression of insulin, *Igf1*, and *Igf2* trophic factors. The higher level of *Igf2* compared with *Igf1* mRNA is consistent with the concept that IGF-2 plays more important metabolic and mitogenic signaling roles during early development, whereas IGF-1 regulates similar functions later in development and life.^29^ The current work suggests that ethanol-associated impairments in placental insulin signaling could be mediated by reduced insulin mRNA expression, akin to trophic factor withdrawal. The selective absence of an ethanol inhibitory effect on IGF-2 suggests that IGF networks may be less vulnerable than insulin pathways to the adverse effects of ethanol. The significant soy-mediated increases in both insulin and IGF-2 in ethanol-exposed placentas suggest that the insulin-sensitizing, anti-inflammatory, and antioxidant effects of soy enhance the availability of trophic factors and ligand regulation of pathways utilized for growth, metabolism, and placentation in the setting of chronic ethanol exposure. In addition, there is supportive evidence that plant-based phytonutrients in soy positively impact metabolism and cellular functions by influencing gene expression.^30^

The expression of insulin, IGF-1, and IGF-2 receptors in placental tissue has been previously reported.^24^ The ethanol-associated inhibition of *Insr* mRNA is consistent with previous findings from ELISA analyses,^1^ suggesting that its expression is regulated at the transcriptional level. However, the reduction in *Igf1r* mRNA level observed with ethanol exposure does not align with immunoreactivity results, which indicated no significant modulation by ethanol. There are no prior data on the impact of chronic ethanol exposure on IGF-2R immunoreactivity. Dietary soy differentially impacted insulin/IGF receptor expression, suppressing insulin receptor mRNA levels in control groups while elevating *Igf1r* and *Igf2r* levels in ethanol-exposed groups. The reduced insulin receptor mRNA levels in both control and ethanol soy groups, compared to casein controls, are consistent with multiplex ELISA findings. However, the comparable levels of *Insr* and *Igf1r* mRNA in the CS and ES groups contrast sharply with the significantly lower protein levels in the ES group.^1^ Altogether, these findings suggest that the expression of placental insulin and IGF receptors is regulated at the mRNA level. However, in the setting of chronic ethanol exposure, post-transcriptional mechanisms, such as translation regulation or protein stability, negatively impact receptor protein expression.

The concurrent reductions in insulin polypeptide and receptor levels in the EC group indicate that ethanol impairs placental insulin signaling, consistent with previous reports in other cell types and tissues.^26,31^ In contrast, the IGF-1 pathway appears to be moderately resistant to ethanol’s inhibitory effects; despite reductions in the *Igf1r* mRNA level, *Igf1* mRNA and IGF-1R protein expressions were preserved. Similarly, the absence of effects on *Igf2* and *Igf2r* mRNA expression reflects the preservation of related pathways vis-à-vis chronic ethanol exposure. A puzzling observation was that despite dietary soy-associated reductions in INSR and IGF-1R proteins, and a lack of *Insr* mRNA stimulation, placentation normalized, and the phenotypic effects of FASD were abolished.^1^ Therefore, while it is reasonable to attribute the ethanol effects to impairments in insulin signaling, the rescue effects of dietary soy were not mediated by the restoration of insulin pathway mediators. Similarly, the findings that *Igf1* mRNA level was unaffected, *Igf1r* was normalized, but IGF-1R protein was inhibited do not strongly support the notion that IGF-1 signaling was restored by dietary soy. Instead, the dominant positive responses were observed with respect to IGF-2, as both ligand and receptor mRNA levels were significantly increased by dietary soy in the ES group. IGF-2 can compensate for impaired insulin pathways by supporting mitogenesis and metabolic functions during development.^32,33^ This study provides the first demonstration that the positive rescue effects of dietary soy during development are mediated, at least in part, through IGF-2-activated networks.

The present study included measurements of *Irs1*, *Irs2*, and *Irs4*, whereas the multiplex ELISAs evaluated in the earlier publication only assessed IRS-1 protein expression.^1^ In the casein groups, the inhibitory effects of ethanol on *Irs1* mRNA level corresponded with decreases in protein concentration. Along with the further demonstration of ethanol-reduced *Irs2* and *Irs4* mRNA levels, it is reasonable to conclude that chronic gestational exposure to ethanol broadly inhibits signaling through IRS molecules, thereby contributing to impairments in placentation and fetal development. Dietary soy’s broad upregulation of *Irs1*, *Irs2*, and *Irs4* mRNA levels in ethanol-exposed placental tissue supports the notion that the corresponding normalization of impaired placental and fetal development was mediated by enhanced signaling through these docking proteins. However, the interpretation of the data is limited by the lack of information on IRS-2 and IRS-4 protein expression. For example, although dietary soy elevated *Irs1* mRNA to levels above those in the EC, CC, and CS groups, IRS-1 immunoreactivity was suppressed relative to the CC and EC groups, suggesting that the capacity to transmit downstream signals through IRS-1 may have remained compromised. Conversely, the lower levels of inhibitory S312 phosphorylation of IRS-1 would have supported IRS-1 signaling in ethanol-exposed placentas.^1^ The lack of information about the abundances of IRS-2 and IRS4 limits further mechanistic interpretation. However, it can be speculated that the combined increases in *Irs1*, *Irs2*, and *Irs4* mRNA levels induced by dietary soy in ethanol-exposed samples contributed to the prevention of FASD and placentation impairments.

ASPH is a downstream target of insulin/IGF signaling.^34,35^ Its stimulatory effects on cell motility and invasion, which are essential for placentation, are mediated by catalytic activation of Notch (hydroxylation) and the subsequent increase in *Hes1* transcription.^4,7,35,36^ Previous experiments demonstrated that molecular silencing of ASPH in the placenta inhibits trophoblast motility, Notch signaling, and fetal growth.^37^ Correspondingly, ethanol inhibition of ASPH expression in the placenta is associated with impaired placentation and IUGR, along with inhibition of Notch-1 signaling.^24^ Therefore, it was of interest to determine if dietary soy positively impacted ASPH expression to mediate enhanced placentation in ethanol-fed dams.

The adverse effects of ethanol on insulin and IGF signaling can be detected by measuring the expression of downstream molecular targets such as ASPH.^2,21,28^ Previous studies have demonstrated that *Asph* mRNA and protein levels are increased through insulin or IGF stimulation.^34^ ASPH is abundantly expressed in normal placental trophoblasts and plays functional roles in placentation, including implantation,^37^ which is crucial to fetal development. Consistent with previous reports, chronic ethanol exposure inhibited ASPH expression. In addition to reducing its mRNA levels, ethanol inhibits ASPH protein by increasing GSK-3β activity, leading to its phosphorylation and proteolytic degradation.^2,35^ The modest but statistically significant increase in ASPH protein in the ES group compared with the placentas from the EC group corresponds with previously reported reductions in GSK-3β activity.^1^ The increased ASPH expression in the placentas of ES dams likely contributed to the normalization of placentation and the prevention of FASD effects.

ASPH functions in part by hydroxylating and activating Notch^37^ within its epidermal growth factor-like domain.^38^ Activation of Notch results in cleavage and nuclear localization of the Notch intracellular domain,^10^ followed by increased transcription of *HES* or *HEY* genes.^10^ Although ethanol did not reduce Notch expression in the EC group, dietary soy significantly increased *Notch1* mRNA in the ES group, correspondingly increasing *Hes1* expression. In contrast, the EC group was associated with significantly reduced *Hes1* expression. Altogether, these findings suggest that dietary soy enhances insulin/IGF signaling through IRS molecules, thereby improving placentation by increasing ASPH expression and Notch activation of HES1.

## 5. Conclusion

The combined information from the earlier report and the present study provides a better mechanistic understanding of how alcohol exposures during pregnancy impair placentation and how dietary soy could ameliorate these adverse effects. Future studies should investigate the extent to which dietary soy interventions at different stages — early gestation, later gestation, or pre-gestation — prevent alcohol-related impairments in placentation and fetal development. Recent experimental evidence highlights the benefits of postnatal and adolescent-stage dietary soy in preventing long-term cognitive and motor deficits caused by chronic ethanol exposure.^21^ The potential benefits of consuming lower levels of soy or other phytonutrient-rich legumes during pregnancy should be studied, as a 100% dietary soy protein regimen would be extremely challenging to maintain. Furthermore, evidence suggests that prenatal dietary soy has beneficial effects on normal brain development and function^1^ and supports insulin resistance disease states^39^ known to be linked to neurodegeneration.^40^

## Figures and Tables

**Table 1.** Summary of ethanol and dietary soy effects on placental expression of insulin, IGF, and IRS signaling pathway molecules

Variable (mRNA)	*F*-ratio	*P*-value
Hormone		
* Ins*	11.36	<0.0001
* Igf1*	10.27	<0.0001
* Igf2*	13.02	<0.0001
Receptor		
* Insr*	5.763	0.0015
* Igf1r*	3.775	0.0141
* Igf2r*	6.74	0.0006
Substrate		
* Irs1*	12.16	<0.0001
* Irs2*	9.356	<0.0001
* Irs4*	47.62	<0.0001

Notes: The mRNA transcripts were measured in CC, CS, EC, and ES placental tissue homogenates through qRT-PCR analysis. Results were normalized to 18S rRNA measured in the same samples. Inter-group comparisons (*n*=8/group) were conducted using one-way ANOVA. The *F*-ratios and *P*-values are tabulated. See Figures 1–3 for graphed data and *post hoc* Tukey multiple comparisons test results.

**Table 2.** Ethanol and dietary soy effects on placental expression of ASPH, Notch1, and HES1

Variable	*F*-ratio	*P*-value
mRNA		
* Asph*	2.431*	0.0726*
* Notch1*	5.692	0.0013
* Hes1*	8.342	<0.0001
Protein		
ASPH	21.63	<0.0001

Notes: The mRNA transcripts were measured in CC, CS, EC, and ES placental tissue homogenates through qRT-polymerase chain reaction analysis. Results were normalized to 18S rRNA measured in the same samples. ASPH protein was measured using ELISA, with results normalized to protein content. Inter-group comparisons (*n*=8/group) were conducted using one-way ANOVA. The *F*-ratios and *P*-values are tabulated.

* Marks *P*-values with a statistical trend (0.05≤ *P* ≤ 0.10). See Figure 4 for graphed data and *post hoc* Tukey multiple comparisons test results.

## Data Availability

Data can be shared on request from the corresponding author.
